# *Helicobacter hepaticus*, a new pathogenic species of the *Helicobacter* genus: Similarities and differences with *H. pylori*

**Published:** 2013-09

**Authors:** Tahereh Falsafi, Mohaddese Mahboubi

**Affiliations:** Department of Biology, Alzahra University, Tehran, Iran

**Keywords:** *Helicobacter hepaticus*, Identification, Metabolism, Virulence factors, Efflux pumps

## Abstract

*Helicobacter hepaticus* was discovered in 1992 as a cause of liver cancer in the A/JCr mouse model. In susceptible mice, infection by *H. hepaticus* causes chronic gastrointestinal inflammation leading to neoplasia. It can also cause morphological changes in breast-glands leading to neoplasm and adenocarcinoma in mouse models. Studies performed on humans have revealed that *H. hepaticus* may also be a human pathogen since infection by *H. hepaticus* can be associated with cholecystitis, cholelithiasis and gallbladder cancer. *H. hepaticus* is a close relative of *H. pylori*, but it lacks the major virulence factors of *H. pylori* including vacoulating cytotoxin A (VacA) and cytotoxin associated gene (*cagA*). Moreover, SabA, AlpA, and BabA, three important adhesin proteins of *H. pylori*, are absent in its genome. In contrast, the genome of *H. hepaticus* contains genes encoding some orthologus virulence factors of *Campylobacter jejuni* such as cytolethal distending toxin (CDT), and PebI adhesin factor. Other genes including 16S rRNA, 18 KDa immunogenic protein, and urease structural subunits are related to *H. pylori*. Its genome contains a small island consisting of 71 Kbp named HHGI1, which probably encodes a secretion system type IV (T4SS), and some other virulence factors. As far as the immunogenic antigens are concerned, the lipopolysaccharide (LPS) and flagellin of *H. hepaticus* are weak stimulants of the immune system, while pro-inflammatory responses are mainly induced by its lipoproteins and most likely by the peptidoglycan. Concerning the multidrug efflux pumps, a homologue of *H. pylori* TolC, HefA, has been observed in *H. hepaticus* which contributes to resistance to amoxicillin and bile acids.

## INTRODUCTION

In 1992, the pathologists at the Fredrick-Institute of the Cancer Research Center, experiencing the effect of chemicals on long cancer in mouse model found that the mice A/JCr serving as negative controls, exhibited an unexpected rate of cancer. Histopathological evaluation of this control group suggested presence of liver tumor in a large number of them. Their food, water and litter were negative for presence of any agent as potential cause of chronic hepatocellular tumors and liver inflammations. Furthermore, the mice that were kept and fed separately also developed liver Pathology. Liver inflammation was more severe in male mice than in females and could be observed in numerous cases of mouse strains except C57BL/6NCr. Moreover, infection could be transmitted to uninfected mice by homogenized liver suspensions. Silver staining of the specimens obtained from the mice livers demonstrated presence of the spiral bacteria in bile and its canaliculi which could grow in microaerophilic conditions (1–2). Later studies have revealed that naturally infected mice can develop a local unpurulent necrosing liver inflammation which progress to active chronic liver inflammation. Development of active chronic inflammation in liver, the mediator of hepatocellular neoplasm in *H. hepaticus* infected mice, has led to the conclusion that this organism is a mice pathogen (3–4). Further analysis has shown that infection by *H. hepaticus* increase nitrogen and oxygen active substances, which lead to an oxidative stress in liver, a process that plays an important role in the liver cancer (2). The investigators have also noted that Tumor Necrosis Factor-α (TNF-α), induced through infection by *H. hepaticus*, have been involved in the development of cancer in abdominal cavity, liver and other organs (5). In addition, several studies performed in mouse models have suggested that *H. hepaticus* can trigger mammary carcinoma. Mechanism, which have been proposed for development of the mammary carcinoma, would be related to the fact that dysregulation of the host immune responses due to infection by enteric bacteria, may induce development of extraintestinal cancers (5).

*H. hepaticus* has received the most attention since it was the first *Helicobacter* sp. that was recognized as a cofactor for the hepatic carcinogenesis. Induction of malignancies in mice after exposure to *H. hepaticus* provides a useful model to study the pathogenesis of infection and generation of the liver cancer in humans. The pivotal roles of the innate immunity cells in colorectal cancer have also been revealed after infection of immunodeficient mice by *H. hepaticus* (6–9).

In human, presence of *H. hepaticus* in the bile samples of patients with cholelithiasis, cholecystitis and gallbladder polyps, has been traced by nested PCR and in situ analysis of the bile samples. Further studies on pathogenesis of *H. hepaticus* have supported the hypothesis that *H. hepaticus* could be a human pathogen and associated with diseases of liver and biliary tract (10–12). Investigaters have also reported that *H. hepaticus* may be a risk factor for the progression of liver disease to cirrhosis and hepatocellular carcinoma, especially among the patients chronically infected with hepatitis C virus (13–14). In addition, higher titers of specific anti-*H. hepaticus* antibodies in patients with gallbladder cancer, compared to the control group, has suggested that *H. hepaticus* infection may be associated with gallbladder cancers in human (15–16). The researchers have also observed that patients with cholelithiasis and cholecystitis associated with gastric cancer had significantly higher prevalence of *H. hepaticus* infection than patients with other diseases (12, 15). Although *H. hepaticus* infection have been observed in 82% of gallbladders and 87.5% of related malignancies, it is not clear whether this organism is causative of gallstone, conducting to malignancy, or contributes as a risk factor (16).

*H. hepaticus* may enter the human body through the contaminated food and water. It can invade the tissues and produce chemical carcinogens that potentially damage DNA. Production of multiple gene mutations may then transform the normal cells into the cancer cells. *H. hepaticus* has also been detected on the intestinal epithelium surface and depth of crypts particularly in cecum. In fact, *H. hepaticas* may be present more frequently in intestine than in liver. Therefore, it is suggested that intestine is the primary site of *H. hepaticus* colonization in human (5, 12, 16).

In this paper we reviewed the literature for the potential pathogenicity of this microaerophilic bacterium and its differences with the human specific pathogen, *H. pyori*.

### General Features of *H. hepaticus*

**Fig. 1:**
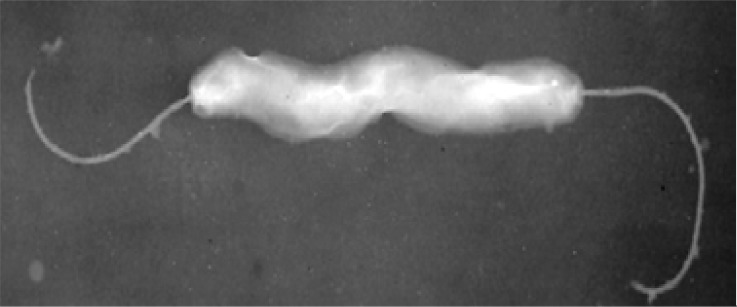
*Helicobacter hepaticus* with bipolar flagella (2).

*H. hepaticus* is a spiral bacterium with 1.5-5 µm length and 0.2-0.3 µm width that is smaller than *H. pylori*. This species can grow in both anaerobic and microaerophilic conditions. Its bipolar flagella are sheathed but contrary to *H. pylori*, lack the periplasmic fibers (Fig. 1).

*H. hepaticus* 51449 ATCC strain contains a circle chromosome with 1799146 bp and its G + C content is 35.9% that is between G+ C% of *H. pylori* and *C. jejuni*. The size of its genome is a little larger than those of *H. pylori* and *C. jejuni*, containing 1875 open reading frame (ORF) that expresses 1875 proteins. Most of *H. pylori* virulence factors, including almost of cag pathogenicity islands, are absent in *H. hepaticus*. Interestingly, the homologues of *C. jejuni cdt* (cytotoxin) and *peb*I (adhesin) genes have been found in its genome (17). There are 9, and 4 chemosensory proteins in *H. hepaticus*, and *H. pylori*, respectively suggesting that *H. hepaticus* interacts with more chemical agents than *H. pylori* for its spatial orientation. *H. hepaticus* lacks the secretion system (comB locus) which is used for natural competition in *H. pylori* (17–18). Evaluation of 16S rRNA gene sequences has shown that *H. hepaticus* is the closest relative of *Helicobacter muridarum* (19).

Similar to *H. pylori*, *hepaticus* is catalase and oxidase positive and rapidly hydrolyses urea. However, it can hydrolyse nitrate to nitrite and can also produces H S. It is resistant to cephalotin and nalidixic acid but susceptible to metronidazole (2). Metabolic capabilities of *H. hepaticus*, and *H. pylori* are likely to be similar however, there are sufficiently differences between their metabolic potentials to provide interesting view of their basic physiology. There is a possibility for the expression of a NADH-1 and NADH-2 dehydrogenase, cytochrome bd and cytochrome *cbb3* terminal oxidase in *H. hepaticus*. So, with high diversity in respiratory system, *H. hepaticus* can adapts with harsh conditions of intestinal tract, liver and gallbladder. Respiratory chain of *H. pylori* with only one NADH-1 dehydrogenase and cytochrome *cbb3* terminal oxidase has the lower diversity than *H. hepaticus* (17). Furthermore, there are important differences between these two species concerning the genes encoding for tricarboxylic acid cycle components. For example, three and four out of the genes that encode five oxidizing metabolite enzymes from α-keto glutarate to oxaloacetate are absent in *H. hepaticus* and *H. pylori*, respectively. Among these enzymes, succinyl-CoA-acetoacetyl-CoA transferase is absent in both *H. pylori* and *H. hepaticus* but the gene encoding malate dehydrogenase is present in *H. hepaticus*, only. This suggests the role of a tricarboxylic acid branch in *H. hepaticus* metabolism, which acts in reductive pathway, a characteristic observed in many anaerobic bacteria (17).

### Identification of *H. hepaticus*

#### Culture and Isolation

**Fig. 2:**
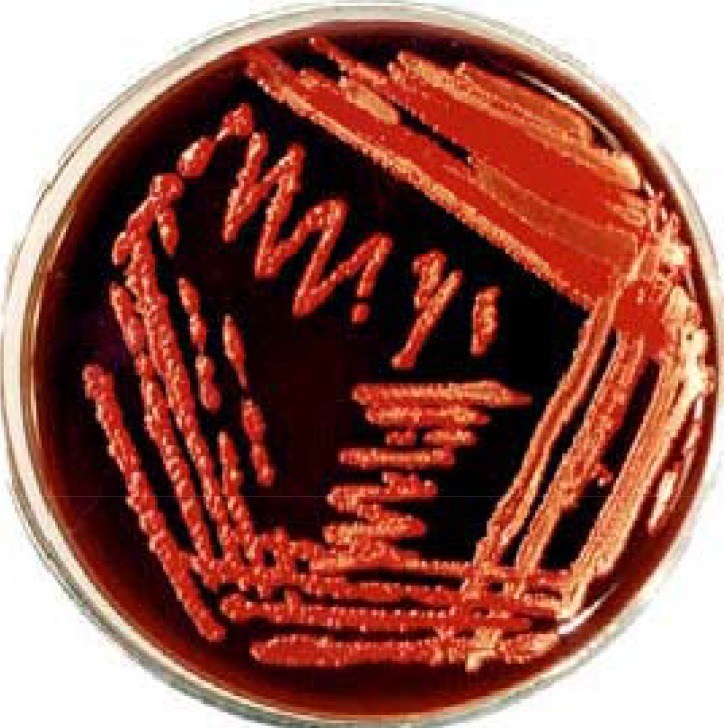
A *H. pylori* strain isolated from biopsy specimen producing the spreading colonies on *Bleu-horizont* agar plate (21).

The samples (feces, biopsy and tissue) can be stored in Brain Heart Infusion broth or Brucella broth containing 30% glycerol at -70°C. To isolate *H. hepaticus*, homogenization of fresh samples in phosphate buffered saline (PBS) and filtration by 0.45 µm filter before cultivation on blood agar containing trimetoprim, vancomycin and polymixin B (11, 20) is recommended. Incubation under microaerophilic condition for a minimum period of 3-7 is also required. Furthermore, it was noted that inoculation of bacteria in Brucella broth with 5% bovine fetal serum and incubation with shaking for 24-48 h would increase its growth speed (11). On culture plates, *H. hepaticus* has been observed as mucoid film or under spreading form without development of the isolated colonies. The experiments have shown that the dilution methods cannot be used for quantitative identification of *H. hepaticus* since it cannot produce isolated colonies on solid medium (11). *H. pylori* produce the isolated small colonies on culture plates however, some strains of *H. pylori* could also generate the mucoid or spreading colonies, as demonstrated in Fig. 2. Unlike other enterohepatic species of *Helicobacter* such as *H. bilis*, *H. fennelliae* and *H. pullorum*, *H. hepaticus* have not yet been cultured from human although its role in inflammatory diseases of human has been demonstrated (11, 20).

#### Histopathology and antibody based tests

It is possible to localize *H. hepaticus* in samples, using Warthin-Starry or Steiner silver staining. Moreover, the application of immunoflourcent rabbit antisera containing polyclonal anti-*H. hepaticus* in liver parenchyma and gallbladder is possible (1, 4).

Commercial serologic tests for diagnosis of *H. hepaticus* infections are not available. However, using cellular particles, membrane digested products or recombinant antigens, the anti *H. hepaticus* specific IgGs have been evaluated by ELISA test (22). Because of cross-reaction between *H. hepaticus* and *H. pylori*, antiserums against *H. pylori* can be used for detection of *H. hepaticus* in mice liver tissue by immunohistochemistry with biotin avidin (1). Commercial kits using polyclonal antibodies against *H. pylori* have been evaluated for specific detection of *H. pylori* in human biological samples such as stool (23). However, for the reason of cross-reaction between *H. pylori* and hepaticus, these commercial antibodies cannot be used for the detection of *H. hepaticus* infection in humans. However, a new monoclonal antibody obtained from hybridoma clone (HRII-51) has shown a high specificity for *H. hepaticus* without any cross-reaction with other gastrointestinal bacteria. Using ELISA, the sensitivity and specificity of this antibody directed against a 15 KDa molecular weight immune reactive antigen have been reported 87% and 97.6%, respectively (22).

#### Polymerase chain reaction

Using the commercial kits conceived for DNA extraction from tissue, *H. hepaticus* have been identified by PCR in scratched samples from cecum (24). Nucleotide sequences of *ureAB* genes have been employed in RFLP and PCR test for identification of *H. hepaticus*. Real time PCR has also been developed for identifying *H. hepaticus* in cecum and fecal samples by detection of *cdtB* gene (25).

### Virulence Factors

#### Cytolethal distending toxin (CDT)

**Fig. 3:**
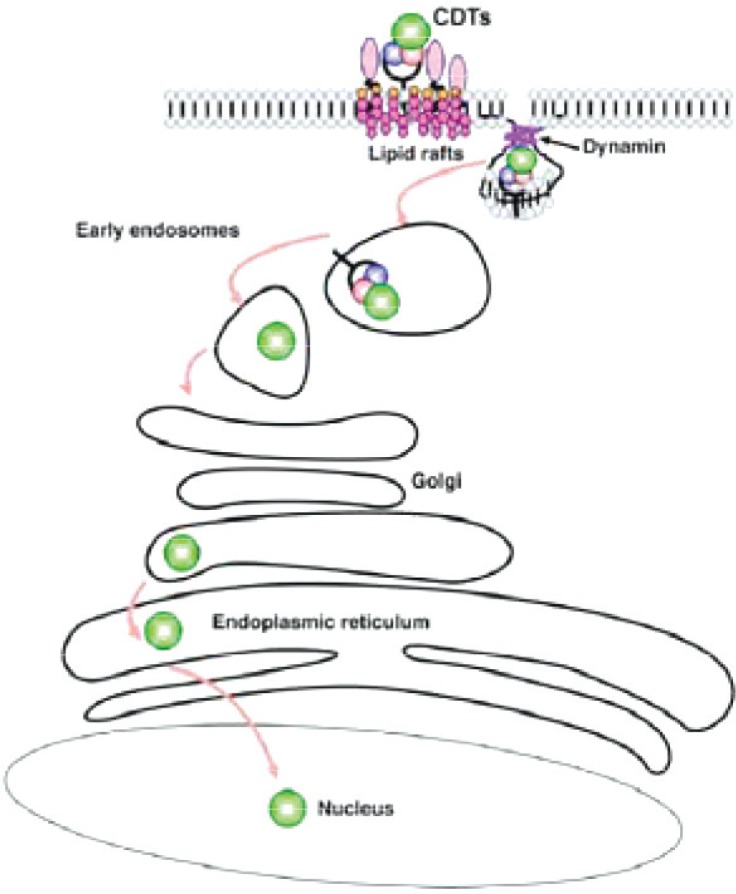
Pathway of CDT entry to the host cell (27).

CDT is a heterodimeric A-B_2_ toxin protein encoded by three neighboring genes namely *cdt*A, *cdtB* and *cdtC*, which are essential for cellular cytotoxicity (20). CdtA and CdtB subunits create non-globular amino acid extensions and these extensions interact with CdtC subunit (26). CdtB is a conserved component of holotoxin in CDT producing bacteria and CdtA is responsible for attaching to the cell membrane, while CdtC helps to transmit CdtB into the nucleus (27). The nature of surface receptor for this toxin is not fully understood but contact of CDT with healthy lipid rafts is needed for its entry via dynamin dependent endocytosis. Fig. 3, schematize the retrograde transmission of toxin via Golgi complex to endoplasmic reticulum and then to the nucleus where its toxic effects may be manifested (27). CdtB is an Mg^+2^ and Ca^+2^ dependent neutral nuclease, containing DNA hydrolyzing and cation binding domains (28). It hydrolyzes the double strands DNA via phosphodiester bounds and creates the mono and oligo deoxyribonucleotides. After entry to target cells, CDT can progressively cause cytoplasm and nuclear extension, and stop cell growth in G2/M phase of eukaryotic cell cycle (28). Thus, by preventing the growth of the infected host cells, CDT helps in the persistence of infection. By its direct and /or indirect effects on T cell and antigen presenting cells, CDT is also able to interrupt the immune response. Therefore, CTD play a principal role in colonization of intestinal tract and increases the severity of mucosal inflammation in the liver diseases of sensitive mice strains (29).

It was also revealed that CDT of *H. hepaticus* plays a principal role in the activation of pre-inflammatory NF-kB pathway during the progress of infectious hepatitis to dysplasia injuries and increases hepatocytes development. Pre-inflammatory NF-kB pathway is activated during the progress of infectious hepatitis to dysplasia injuries; therefore, CDT may have the carcinogenic potential in vivo conditions (30). Induction of apoptosis throughout *H. hepaticus* infection was also observed and apoptotic bodies are formed in the last apoptotic stage, which can induce anti-inflammatory cytokines and reduce the pre-inflammatory cytokines for facilitating immune inhibitory effects. Apoptosis could be induced via two major pathways: Extrinsic pathway that starts with activation of death receptors while intrinsic pathway is activated with a change in mitochondrial membrane potential. Experiments have revealed that contact of the INT407 cells with CDT activates caspase 3, 7, 9, suggesting involvement of mitochondrial apoptosis in the case of *H. hepaticus* infection (31).

Other in vitro cytotoxic activity of CDT is granulating activity that is different from the activity of VacA in *H. pylori* (29). There is no *cdt* gene in *H. pylori* but, regarding its mechanism of action, CDT may be an equivalent gene of VacA as immune regulatory toxin in *H. hepaticus*.

#### Urease

Like as *H. pylori*, hepaticus expresses a multimeric urease enzyme that uses nickel as a cofactor. In *H. pylori*, active urease hydrolyses the urea into ammonium and bicarbonate, which protects this bacterium from the gastric acidic microenvironment and acts as a nitrogen source. *H. hepaticus* is sensitive to acid and addition of urea into the cultured medium, cannot protect the bacteria from acidic pH 3. Therefore, *H. hepaticus* urease is not involved in resistance to acid. Urease gene cluster of *H. pylori* and *H. hepaticus* contains structural genes (*ureAB*) and accessory genes (*ure IEFGH*) however, the distance between *ureI* and *ureB* in *H. hepaticus* is 9 bp smaller than that of *H. pylori*, suggesting that there is no promoter in upstream of *ureI* in *H. hepaticus*. This fact limits the probability of regulation at transcription level or afterwards (32–33).

In *H. pylori*, urease system is induced via nickel at transcription level; it is dependent on nickel and regulatory protein NikR that regulates the nickel adsorption via another gene, namely nixA (32).

Presence of nikR in *H. hepaticus* genome has been demonstrated, but its role in the regulation of urease was not quite determined. It was proposed that NikR affects independently urease activity, via regulation of nickel adsorption. In addition, the urease system of *H. hepaticus* lacks a homologue of nixA although; an ABC transporter gene was detected near its urease operon that may be involved in nickel uptake. Therefore, unlike *H. pylori*, regulation of urease via nickel in *H. hepaticus* may not be at transcriptional level. It is proposed that this regulation may at translational level, presumably via activation of urease apoenzyme, like the case of *Streptococcus salivarius* (33). It should be noted that enteropathogenic *Helicobacter* species of rodent's intestinal tract does not require the high level of urease activity but there is a high level of urease activity in gastric *Helicobacter sp*. Therefore, Enteropathogenic *Helicobacter* sp. use urease system for nitrogen metabolism and ammonium store, only.

**Table 1:** General features of *H. hepaticus* genome (17).

Total size	1, 799,146
GC content,%	35.9
Coding sequences	1,875
Average gene length, bp	1,082
Coding density,%	93,04
Predicted secreted proteins	347
Predicted membrane proteins	358
Predicted proteins with assigned function	1,022
Ribosomal RNA	1×16s-23s-5s
tRNA	37 (7clusters, 15 single genes)

#### DNA binding protein from starved cells (DPS)

DPS is a member of ferritin like proteins, identified in *H. hepaticus*. The mutant of *H. hepaticus* lacking DPS cannot grow in conditions with 3% oxygen. It is more sensitive to oxidative reagent such as H_2_O_2_, cumene, hydroperoxide and t-butyl hydroperoxide and has more damaged DNA, which can lead to lysis or change to coccoid form (34). Pure DPS protein from *H. hepaticus* is able to bind to both iron and DNA, and compared to the natural DPS or DPS without iron, the DPS-iron form has higher ability to attach to DNA. Phylogenetically, *H. hepaticus* DPS protein is relative to *H. pylori* NapA protein. DPS and NapA act by scavenging of irons and protect the bacterial DNA under oxidative conditions. Therefore, in absence of iron, DPS proteins are oligomerized (34). N-terminal of DPS, at its first N-terminal α-helix, is rich in lysine and plays an important role in attachment of DPS to DNA (35).

#### Catalase

Chronic infection of mice with *H. hepaticus* has been distinguished by infiltration of neutrophil and macrophages, which lead to produc-tion of reactive oxygen intermediates in cecum or liver. The free radicals of oxygen, secreted during infection may increase the damage of DNA in intestinal hepatocytes or epithelial cells that leads to colitis or hepatitis. To circumvent these substances, *H. hepaticus* produces a catalase that may be cytoplasmic or periplasmic. Its periplasmic location is similar to that of other Gram-negative bacteria such as *Pseudomonas syringae*, *Brucella abortus* and *Vibrio fischeri* (36). Periplasmic location of catalase in *H. hepaticus* and other Gram-negative bacteria facilitate the antigenic presentation to mammalian immune system and mediate the immune responses. Therefore, the catalase of *H. hepaticus* acts as immunogenic target since host can differentiate this catalase from the endogen one (36).

#### Pathogenicity Islands (HHGI1)

The genome of *H. hepaticus* contains a large and some small regions with a different G + C content suggesting the horizontal transfer of latter region. The largest region encodes the proteins including three proteins with homology to structural components of type IV secretion system. Unlike many other pathogenicity islands, HHGI1 of *H. hepaticus*, is not interspersed by the direct repeats and does not have any gene for tRNA. Furthermore, there is an integrase gene like the integrates gene of P4 (HH269) that is present in pathogenicity islands. Existence of secretion systems and other secretary proteins suggest that HHGI1 would be the true pathogenicity islands however, these genomic or pathogenicity islands are not present in all strains of this species. Different studies have suggested that the strains containing HHGI1 are more virulent than the strains lacking these regions (17, 37).

#### Flagella

*H. hepaticus* is a spiral bacterium with a bipolar-sheathed flagellum. This filamentous structure is composed of two flagellin subunits, FlaA and FlaB. In relative bacteria, flagellin genes are mainly regulated by sigma factor FliA (σ^28^). *H. hepaticus* has two similar copies of the *flaA* (*flaA1* and *flaA2*) genes that encode major subunits of flagellin FlaA. Inactivation of each copy of these *flaA* genes has small effect on flagellum morphology and expression of *flaA*, however; inactivation of *flaA*-1 has a more prominent effect on the motility of bacterium (18). Mutations in two genes of *flaA* or in *fliA* cease FlaA synthesis or produce small flagella; these mutants cannot colonize the mice (38). Genetic documents suggest that the components and regulatory genes of *H. hepaticus* flagellum are completely relative to *H. pylori*. In addition, like those of *H. pylori*, the flagellar genes of *H. hepaticus*, are distributed throughout the bacterial chromosome. In *H. hepaticus*, flagella are the important antigenic targets for innate and adaptive immune systems. Moreover, its hook protein, FlgE is a T-cell dependent dominant antigen (38–39).

#### Adhesins and outer membrane proteins

The factors involved in colonization and virulence of *H. hepaticus* are not similar to those of *H. pylori*. Most of *H. pylori* adhesin proteins including *sabA*, *alpA*, and *babA* are absent in *H. hepaticus*. *H. hepaticus* carries 11 genes that encode the proteins homologue to a large family of outer membrane proteins (17). This family consists of 33 analogous genes classified into two subfamilies; Hop and Hor. The adhesins of *H. pylori*, *BabA*, *SabA*, *AlpA*, *AlpB* and *HopA-E* may be considered as prototypes for Hop family. This subfamily has a sectile amino terminal motif while Hor proteins with unknown function lack this motif. Slight comparison of *H. hepaticus* outer membrane proteins with other outer membrane proteins, provides no clear result about their functions (17). None of Hop proteins in *H. hepaticus* contains the typical Hop protein amino terminal motif. Phylogenetically comparison of outer membrane, either Hor, or Hop proteins of *H. hepaticus* with porins of *E. coli* (HH0525, HH1713, HH0661, HH1453, HH0812), suggests that these proteins may be the porins. A few OMPs of *H. hepaticus* are related to Hor proteins such as Hor G of *H. pylori*. In general, there is no significant similarity between outer membrane proteins of *H. hepaticus* and those of *H. pylori*. *H. hepaticus* does not colonize the human gastric epithelium and among its proteins involved in the attachment to epithelial cells, one protein (HH1481) demonstrates 72% homology with PebI of *C. jejuni*, a protein not found in *H. pylori* (17, 20).

#### Lipopolysaccharide (LPS)

LPS and its lipid A, is not well studied in *H. hepaticus* however, the lipid content (small chains of fatty acids) of *H. hepaticus* is different from that of H. pylori. Among *Helicobacter* sp., LPS of *H. hepaticus* and enterohepatic *Helicobacter* sp. display lower activity in limulus amebocyte assay (39). In susceptible mice, *H. hepaticus* can escapes or inhibit innate immune response of gastric epithelium (18). It is revealed that bacterial lysate and especially soluble components of LPS disturb innate immune responses via TLR4, and TLR5. Inhibition of innate immune responses by *H. hepaticus* LPS, can affect the responses to resident microbial flora, epithelial homeostasis and inflammatory conditions of intestine (39).

#### Antibiotic-resistance and efflux pumps

Resistance to commonly used antibiotics is frequent in the case of *H. pylori* infection (40–41). *H. hepaticus* is able to resist to antimicrobial agents but contrary to *H. pylori*, it is also resistant to bile acids. Multiple studies on the mechanisms of resistance to antibiotics have shown that multidrug efflux pumps are involved in the resistance of *H. pylori* to structurally unrelated antibiotics (42–44). A homologue of *H. pylori* TolC, named *HefA*, have been observed in *H. hepaticus*, which is involved in the resistance to amoxicillin and bile acids (45). An outer membrane protein that is a component of resistance-nodulation-cell division (RND) family is involved in multidrug efflux of antibiotics in *H. pylori*. A difference between *H. pylori* TolC and its homologue in *H. hepaticus* is that, in *H. pylori*, TolC is not involved in resistance to amoxicillin (45). Two other genes, HH0174, and HH0175, are identified in the genomic sequence of *H. hepaticus* that are homologous to inner and periplasmic proteins of CmeABC in *H. pylori*, which may be involved in resistance to bile, macrolide and tetracycline. So, resistance to bile in *H. hepaticus* may be regulated by both hefA and CmeAB orthologus may be regulated by both hefA and CmeAB orthologus (45, 46).

#### Treatment

Different treatment regimens for eradication of *H. hepaticus* infection in mice are described but in many cases, treatment has not been successful although *H. hepaticus* may be sensitive to many antibiotics. Two weeks treatment with amoxicillin via drinking water has not been effective in elimination of infections (41). Prescription from drinking water is less effective than that of gavages since *H. hepaticus* was isolated from the mice that were treated via drinking water. Treatment regiments composed of three drug including combination of amoxicillin, metronidazole and bismuth administrated three times by day for two weeks was more effective via gavages in non-immune mice (6-8 weeks-old). However, this difficult method may limit the success of therapy. In general, contradictory results were obtained concerning the effectiveness of the different regimens. For example, in one study, treatment with four drug regimens including amoxicillin, metronidazole, clarithromycin and omperazole was not able to eradicate *H. hepaticus* and the role of amoxicillin was not definite in this treatment regiment (47–49). Also, treatment of naturally infected mice (8-10 weeks-old) suggested that single dose of amoxicillin, metronidazole and tetracycline was not able to eradicate infection of intestinal tract but amoxicillin or tetracycline in combination with metronidazole and bismuth for two weeks was effective in eradication of liver, cecum and colon infection (47). Today, three drug regimens (inhibitor of proton pumps, amoxicillin and clarithromycin or metronidazole) that are frequently recommended for eradication of human *Helicobacter* infection are also used for treatment of *H. hepaticus* infection in mice. This regiment includes three drug administration including metronidazole, amoxicillin/tetracycline and bismuth for two weeks in mice A/JCr (47–49).

## CONCLUSION

*H. hepaticus* is a Gram-negative bacterium with pathogenicity in mice and humans. Although it has some similarities with *H. pylori*, but shares some characteristics with *C. jejuni*, suggesting that some of its genes may have been acquired from *C. jejuni*. This would be consistent with the suggestion that the intestine is the primary site of *H. hepaticus* colonization in humans. Since this bacterium is implicated in human associated diseases such as gallbladder cancer, cholecystitis, cholelithiasis and other yet unidentified diseases, research pertaining to this field will be of utmost importance in this region as well as in other parts of the world.
